# *‘The law is very, very outdated and not keeping up with the technology’:* novel forms of assisted gestation, legal challenges, and perspectives of reproductive rights advocates in England and Wales

**DOI:** 10.1093/jlb/lsad027

**Published:** 2023-11-01

**Authors:** Elizabeth Chloe Romanis

**Affiliations:** Centre for Ethics and Law in the Life Sciences, Durham Law School, Durham University, Durham, United Kingdom; Petrie-Flom Center for Health Law Policy, Biotechnology, and Bioethics at Harvard Law School, Cambridge MA, United States; Edmond and Lily Safra Center for Ethics, Harvard University, Cambridge MA, United States

**Keywords:** assisted gestation, artificial placentas, assisted reproduction, parenthood, uterus transplantation

## Abstract

A growing body of literature examines the ethico-legal challenges resulting from novel forms of assisted gestation like uterus transplantation and artificial placentas (also known as ‘artificial wombs’). However, there has not yet been consideration of reproductive rights organizations/advocates’ understandings of novel forms of assisted gestation and their challenges. These perspectives provide critical insight into how novel procreative practices are understood and the problems and pressures that might arise from their use. This is the first legal article to engage with reproductive rights organizations/advocates and thus it provides important contextual grounding to existing scholarship about assisted gestation. Focus group discussion epitomized the need for legal reform in key areas surrounding reproduction. Themes were constructed that exemplify what participants highlighted as critical: the need to re-evaluate the fundamentals of legal parenthood, consideration of how novel technologies could further enable the policing of gestation, and the space and time needed for law-making.

## I. INTRODUCTION

For persons unable to sustain a pregnancy, whether for biological, social, or psychosocial reasons, assisted gestation can enable them to reproduce. Surrogacy has long been possible, but novel practices/technologies that may be alternatives to surrogacy are on the horizon. Uterus transplantation enables people without a uterus to undertake gestation and pregnancy.^1^ Artificial amniotic sac and placenta technology (‘artificial placentas’)^2^ might soon enable people to opt out of gestation because a device can take over performing gestation—this is known as partial ectogestation.^3^ There is also speculation about the possibility of artificial placentas one day being capable of facilitating a complete gestation outside of the body (complete ectogestation).^4^ In fundamentally altering the conditions in which gestation may occur (in bodies that did not have a uterus or entirely outside of bodies), novel forms of assisted gestation pose considerable challenges for the law. Through reflexive thematic analysis^5^ of data generated from two semi-structured focus groups, this article explores the views of the people involved in campaigning for changes to the law surrounding the regulation of reproduction; individuals working (or having worked) with or for reproductive rights organizations in England and Wales.

To my knowledge, there had not, before this study, been any research conducted exploring what British reproductive rights advocates’ attitudes to uterus transplantation and ectogestation are, or what they anticipate as the principal challenges arising from their potential use. These perspectives provide critical insight into how novel procreative practices are understood, how they may be used, and the problems and pressures that might arise from their use. Study participants had experience of how law surrounding reproduction operates on the ground (and its impacts), and the political realities of law reform. Further, participants had first-hand awareness of the experiences of different people who cannot gestate or who have experienced a range of difficulties during gestation and birth. Therefore, their insights are invaluable in highlighting how individuals may want to access and use the technologies/practices and inadequacies in the law based on current experiences. This study provides important contextual grounding to the existing scholarship on the legal challenges raised by novel forms of assisted gestation, which at present is doctrinally focused. This article reveals challenges that are currently underappreciated and reinforces existing observations in the literature from a distinct perspective. This study can better help inform future policy/reform efforts in future by enriching the existing speculative literature.

Assisted gestation encompasses technologies/processes^6^ involving some intervention that allows people who are unable (or potentially unwilling) to gestate to reproduce using their own (or donated) genetic material. This can either take the form of undertaking gestation themselves, with some assistance provided by donation of a crucial organ (uterus transplantation), or having another person assist with gestational labor (surrogacy), or having an artificial placenta undertake that labor (ectogestation).^7^While novel forms of assisted gestation may sound like science fiction, they are not. There have been over 30 babies born from pregnancies following uterus transplantation,^8^ with ~100 transplants having been performed worldwide.^9^ The first uterus transplantation in the UK (in England) took place in August 2023.^10^ There are several working teams of fetal scientists that have developed proof of concept models for an artificial placenta.^11^ The devices currently in development are primarily those capable of *partial* (rather than complete) ectogestation, and are designed as an alternative to conventional neonatal intensive care given its limitations, in that conventional care cannot support entities born without sufficiently matured lungs and is associated with high risk of infection.^12^ The artificial placenta ‘represents a novel approach that aims to maintain the innate fetal circulation while promoting normal prenatal development’.^13^ Artificial placentas work by attempting to emulate gestation in artificial conditions: a sealed bag of warmed amniotic fluid, cannula that act as an umbilical cord (to deliver nutrients and remove waste) and a pumpless oxygenator circuit simulate uterine conditions.^14^ With animal testing consistently yielding positive results,^15^ some of the working teams have indicated their intention to move to human testing in the not-so-distant future (one team’s device has been described as ‘nearing clinical application’,^16^ and they have obtained financing for clinical trials).^17^ There are ethical issues remaining in the design and development of artificial placenta devices,^18^ in testing of the processes of extraction on pregnant people, and subsequently, the effect of artificial placenta on developing human entities.^19^ While these issues are the most pressing, there is also value in considering some of the ethico-legal challenges that might arise if and when such devices are introduced as an alternative to conventional neonatal intensive care, or even as an alternative to bodily gestation in its entirety.

Novel forms of assisted gestation, alongside surrogacy, raise complex and distinct issues from other reproductive technologies that alter the process of conception in procreation. While assisted conception raises some complex ethico-legal questions about what human entities come into existence and who they may be genetically related to, assisted gestation raises issues in the generative process of creation that entity is subjected to—including who or what is responsible for the labor involved.^20^ While there is a need for reform of the entire system of regulation surrounding reproduction, in this article I focus on the assisted gestation specifically because the challenges raised to the legal framework are distinct and, as they relate to novel technologies/practices, are underexplored.

In this article, I explore three themes constructed within data generated by focus groups with reproductive rights advocates in England and Wales. First, *re-evaluating the fundamentals of legal parenthood* explores how participants felt that novel forms of assisted gestation pose a challenge to the existing scheme of regulating surrounding parenthood. This involved discussion of novel complex procreative arrangements in which there are competing claims to parenthood and discussion of the cis-heteronormative language used to describe gestation and motherhood. Second, *resisting the policing of gestation* examines participants concerns about how assisted gestation could be policed by law and policy in terms of who is enabled to access novel technologies and who is excluded and how pregnant people’s choices are (not) respected. Finally, *making space and time for law-making* reflects on how participants saw processes of law reform and their thoughts about how this can make use of speculation/forward thinking as well as the complicating factors in implementing a comprehensive and proactive scheme of regulation for technologies not yet in regular use.

## II. METHODS

There has been little empirical work about the regulation of assisted gestation.^21^ To my knowledge, there had, before this study, been no research conducted exploring what British reproductive rights advocates’ attitudes to some of these new technologies are, or what they anticipate as the principal challenges in responding to their development and potential use. Empirical research with individuals working with or for reproductive rights organizations is key because they have an awareness of the way the law surrounding reproduction works on the ground, and the political realities of making changes to the law in this area. Further, these individuals (those working, or who have worked with or for these groups) have first-hand awareness of the experiences of different people who cannot gestate or who have experienced difficulties during gestation and birth. Therefore, their insights are invaluable in highlighting how individuals may want to access and use the technologies/practices. Furthermore, these individuals have considerable insight about any (potential) inadequacies in the law based on current experiences. That said, it is also important that future studies engage with the views and experiences of (potential) service-users themselves (more on this later) and/or the healthcare professionals who attend to them. Data generated from conversation with reproductive rights advocates can better inform some of the speculative doctrinal and socio-legal research currently being undertaken about new forms of assisted gestation, specifically in that it can help aid appropriate focus if the objective of such research is anticipating and thinking about solutions to real problems that might arise in the advent of new technologies.

This study comprised of two 2-hour focus groups (total *n* = 11, with 5–6 participants in each group).^22^ Focus groups allowed me to observe the participants engaging in ‘collective sense-making’—how different perspectives were brought together and ideas are generated between them, and how these ideas were expressed, modified, or changed during interactions.^23^ Participants were recruited through personal networks using purposive sampling to ensure a range of expertise. Some of this expertise was from working for/campaigning with an organization with a focus on one aspect of reproduction eg, fertility, surrogacy, or birthing. Other individuals worked for broader causes intersecting various aspects of the reproductive process eg, promoting public education about reproduction or equality in birthing and parenting for marginalized groups. Though most participants were affiliated with organizations, not all were. Some participants worked, or had worked, in policy or research roles for these reproductive rights organizations. Others identified as activists affiliated with, or who had been affiliated with, reproductive rights organizations. Some identified as having been service-users of assisted reproductive technologies during the focus groups and this comes through in the data, but they were not asked this directly. I did not collect demographic information about participants and their current roles to preserve their anonymity. All participants were primarily based in England and Wales.

The small sample size reflects the niche field from which I was recruiting, but I did recruit from some of the most active organizations in the jurisdiction. Despite limited statistical-probabilistic generalizability (a criticism sometimes aimed at qualitative research) the results have significant analytical generalizability.^24^ My results have the potential to significantly enhance the *conceptual* understanding of the legal challenges resulting from novel forms of assisted gestation in England and Wales.

Both focus groups were semi-structured. At the start of each group, I provided participants with a short verbal explanation of uterus transplantation and ectogestation. Discussion then centered around open-ended questions based on my previous desk-based research.^25^ These questions were broad and open-ended to ensure that the topic of the discussion in each focus group was directed by the participants themselves. The topic guide I used included questions like:

- Do you think that new technologies (uterus transplantation/artificial placenta technologies) will have some unique benefits? In what way?
- What do you think are the biggest problems with the law surrounding reproduction at the moment?
- Do you think that people will see these technologies/practices (artificial placentas; uterus transplantation; surrogacy) as different options on a spectrum? Are they ‘alternatives’?

I encouraged participants to discuss ideas among themselves rather than with me. However, I was not a completely passive moderator; I asked questions to spark discussion, asked for clarification, and sometimes encouraged participants to respond to ideas raised by another participant. At times, I felt myself stifling the inclination to become a participant to the conversation because I felt like ‘one of them,’ having had familiarity with some participants and sharing many of their interests. This has undeniably influenced my interpretation of the data.

The approach to data analysis was based on Braun and Clarke’s account of reflexive thematic analysis, consisting of (i) familiarization, (ii) inductive coding, (ii) generating themes, (iv) developing themes, (v) refining and naming themes, and (vi) writing up.^26^ I used NVivo to undertake inductive coding of the group transcripts. What I identified as codes and how I labeled them is inevitably subjective.^27^ I then began organizing codes into themes without software, which was difficult since this was a rich data set involving intersecting issues. Organizing took several months, which allowed the final themes to reflect my deeper understanding of the data.^28^ The themes constructed each tell a story of ‘particular patterns of shared meaning across the dataset’.^29^ The name of each theme evolved as key messages were refined. In writing up, I contacted participants for their pronouns and a pseudonym. One participant did not respond and was assigned a name.

Aside from what is reported below, there were two other major themes constructed from this study, which I explored in an article elsewhere. These themes—related to the equality-enhancing potential of novel forms of assisted gestation, and the limitations on that potential arising from extra-legal barriers impacting access for marginalized persons—were explored elsewhere because they needed considerable space to be fully developed.^30^ Moreover, those reflections were not related to legal challenges, whereas what is considered in this article relates very much to reflections about the legal framework in England and Wales (though they also have broader relevance). Below, I consider three further themes from this study that relate to the future of regulation or how we should think about and approach future regulation: (i) the need to re-evaluate the fundamentals of legal parenthood, (ii) concerns about the policing of gestation, and (iii) space and time for law-making.

## III. RE-EVALUATING THE FUNDAMENTALS OF LEGAL PARENTHOOD

Where reproduction is assisted, the attribution of parenthood is determined by the Human Fertilisation and Embryology Act 2008 (HFE Act 2008). The HFE Act 2008 specifies that only two persons can be recognized as the legal parents of a child in England and Wales.^31^ It is a long-standing legal principle that motherhood is attributed solely by gestation. As famously put by Lord Simon in the *Ampthill Peerage Case* ‘[m]otherhood, although a legal relationship, is based on a fact, being proved demonstrably by parturition’.^32^ Irrespective of how conception occurs, it is the person who births that is legally defined as ‘mother’.^33^ This maxim has been codified in the HFE Act 2008, which specifies that ‘the woman who is carrying or has carried a child as a result of the placing in her of an embryo or of sperm and eggs, and no other woman, is to be treated as the mother of the child’.^34^

The determination of the second legal parent is more complex. The common law presumption is that the husband of a person who has birthed will be the legal father.^35^ Where the gestator is unmarried/not in a civil partnership, the father is the person named on the birth certificate, or the other genetic progenitor (provider of sperm).^36^Where a person has received fertility treatment in a licensed clinic (and thus the HFE Act 2008 applies), a sperm donor cannot rely on their donation to claim legal parenthood of a resulting child.^37^ The father is the person who consents, and to whom the pregnant person consents to be the father, where the agreed fatherhood conditions in the HFE Act 2008 are met.^38^ This person is the father whether they are married to the gestator or not and irrespective of whether they are the genetic father. The HFE Act 2008 also recognizes a second female parent in the same circumstances: through civil partnership/marriage or their consent and the person receiving the treatment’s consent to them being treated as the second female parent.^39^ The Gender Recognition Act 2004 specifies that ‘[t]he fact that a person’s gender has become the acquired gender under this Act does not affect the status of the person as the father or mother of the child’.^40^

Participants in both focus groups identified the attribution of parenthood and the birth registration system as the greatest issue surrounding reproduction in England and Wales, which needs reform:

Olivia: *“Legal parenthood, bam. What would I pick* [for reform] *from the last 20 years and everything? Legal parenthood.”*

***

Richard: *“I think, if I was to pick one area that needed reform, that should be reformed, that affects everything we’ve talked about, it’s probably how… Well, it’s what we would call today the birth registration process I guess.”*

Hannah was more specific in identifying the biggest challenge as reform of *“motherhood, like, as understood by the law*.” There has been a considerable, and growing, literature critiquing the attribution of parenthood, specifically looking at surrogacy. In England and Wales, the surrogate is the legal mother. If the surrogate is married, her husband will be recognized as the legal parent from birth, unless it is shown that he did not consent.^41^ The intended parents can apply for a parental order to become parents of the child – but they must meet specified conditions.^42^ Criticism has focused on the failure of the law to attribute parenthood in a way that matches the lived experience of involved parties.^43^ As the result of a long process, which found its roots in the academic critique and much campaigning, a recent report published by the Law Commission and Scottish Law Commission recommends a new pathway to legal parenthood, which would recognize legal parenthood at birth for (some) intended parents.^44^ There are some limitations on who the new pathway is accessible to: among other things, there must be a genetic link between the intended parents and the child born through surrogacy, the intended parents must be married/in a civil partnership or in an enduring family relationship, the surrogate and at least one intended parent must be domicile in the United Kingdom, and the surrogate must not have exercised their right to withdraw consent to the intended parents being the legal parents from birth.^45^ The Law Commission reports that over two thirds of their recommendations are implemented into law,^46^ however, surrogacy reform is a complex political issue in this jurisdiction and it is likely that the law surrounding parenthood may remain unchanged for some time.

Participants described the problems with the mechanisms by which legal parenthood is attributed as resulting from new technologies and shifting societal practices surrounding reproduction fundamentally changing the way families are formed. Consequently, the law, and the fundamental principles underpinning the law, are outdated.

Ali: *“I think the current laws around parenthood, or a recognition of parenthood, just predate all the technologies we’ve had in the second half of the 20th century onwards. I mean it’s really archaic… In fact, it’d probably date back to the 19th century or earlier in terms of concept and implementation, it would seem. So, it’s a real muddle legally, and I know in organisations like ours and our kind of community, we operate with completely different language from the way the law operates in terms of how we talk about surrogates, intended parents, who are the parents at birth, the difference between legal parenthood and genetic parenthood and so on.”*

Here, Ali clearly indicates that organizations supporting people using procreative technologies have adapted (in what ways they can) despite the law being far behind.^47^

The remarks thus far are rather sweeping and related to reproductive technologies broadly; they set the context for more specific discussion of novel forms of assisted gestation. Both focus groups reflected on the ways in which *assisted gestation* specifically, and in different forms, could be a potential trigger for the re-evaluation of the legal framework.

In both focus groups participants discussed how novel forms of assisted gestation are, in theory, disruptive to social and legal understandings of parenthood. As Jackson has observed that ‘while we must not lose sight of the significance of social change for the meaning of parenthood…. new technological developments are capable of redefining some of the basic facts of reproduction’.^48^ There are ways in which both uterus transplantation and artificial placentas raise some fundamental questions about the operation of the current law. As Olivia remarked, in the case of an entity being gestated outside of the body, there might be complex questions in trying to apply the law that has, for some time, been maintained on the grounds of legal certainty: *“…so hang on how does the legal definition of the mother apply here?*” Where there is *partial* ectogestation (so an artificial placenta takes over the gestation of an entity that was being gestated by a pregnant person) the definition of the legal mother might be straightforward—it would be the pregnant person.^49^ This, however, would be more complex in the case of *complete* ectogestation (where there is no pregnancy) were it ever possible. Alghrani observes that an artificial placenta clearly cannot be a ‘mother’.^50^ In the absence of law reform, it is hard to see how the law will respond since there is no clear answer to how parenthood will be attributed.^51^

In relation to the difficulties of attributing parenthood, participants also discussed uterus transplantation:

Hazel: *“…there is nothing that would complicate around parenthood* [in uterus transplantation] *because it is the person who delivers, who is the mother-”*

Olivia: *“And is actually a nice, easy-”*

Alex: *“So it is actually very simple-”*

Olivia: *“Unless we are talking of trans persons.”*

Although identifying the legal mother could be considered straightforward (the person who birthed),^52^ there are imaginable instances where the law is confusing. Horsey has explained that ‘if uterine transplants ever become a real possibility, this will raise different kinds of biological issues, and even more so if pregnancy can ever be established in a [trans woman or a cis-gendered-] man’.^53^ To date, uterus transplantation has only been performed in people with physiology assigned female at birth (AFAB), however, several teams of surgeons have indicated that while performing the surgery in people assigned male at birth (AMAB) may be physiologically more challenging—it may not be impossible, and they anticipate such surgeries being performed in the future.^54^

There are two conceptual challenges to the legal framework within all the observations detailed thus far. The first set of challenges concern the values used to determine who is identifiable as a parent following uterus transplantation/ectogestation. The second challenge lies in addressing the gendered and heteronormative language and assumptions within the framework. These two challenges are explored in greater detail below. As the participants observations about both of these challenges illustrate, novel forms of assisted gestation have a fundamental impact on the regulation of reproduction and uniquely raise these two legal challenges because at present, gestation is at the center of how the law determines parenthood and is a key part of how the law genders pregnant people and people who have birthed.

### III.A. Identifying the Parent(S)

Participants explored the attribution of parenthood in surrogacy noting *‘how utterly ridiculous that situation is’* (Olivia) in that it neglects the intentions of putative parents and surrogates. There was a sense, however, that these issues could be ignored within the current legal framework because surrogacy is marginalized in the contemporary climate. Most people who gestate and birth do so because they intend to parent the resulting child. There was a feeling among participants that novel forms of assisted gestation mean we *must* address the fundamental questions about the nature and value of parenthood that, despite their contemporary relevance, legislators have avoided:

Ali: *“It feels like, you know, that needs to be looked at, disentangled, and made fit for purpose and to be resilient to future change. It’s a real mess, and I think other countries have done work around this. You know, it’s not that it’s not possible to do, but we seem to be in a bit of a mess about it. I know surrogacy law is being examined at the moment for reform, and I know that bodies swerve this issue of parenthood and recognition and, kind of, said, ‘Oh, that’s something we need to deal with separately in another legal project.’”*

Participants explained that it is hard to ignore the big questions as technology continually enables different gestations, and gestations that are more visible—this makes the need to interrogate the value we attach to different dimensions of the attribution of parenthood more visible.

Ali: *“it’s really about how we recognise the origins of a child, in terms of its genetic origins, its gestational origins, and its legal parents who then bring us up. And I think, if that was fixed and made futureproof, if you like, that would support a lot of the things we’ve talked about today.”’*^55^

As Ali acknowledges, there are multiple dimensions to how we recognize the legal parent of a child depending on what technology is used, and that only gets increasingly complicated with innovative technologies/practices shifting the nature of gestation. Thus, we must turn to the big questions:

Richard: *“What does gestation mean with respect to parenthood? What does genetics mean with respect to parenthood and what does parenthood mean outside of those two things?”*

As Richard’s way of asking the questions here illuminates, recognition of the shifting nature of gestation (in novel forms of assisted gestation) is the gateway to asking these fundamental questions. First, in that it forces us to ask what a gestational contribution is when, until these novel forms of assisted gestation, this is something that is consistently been taken as a given both socially and legally. Second, where gestation is uprooted as the single most determinative factor in attributing parenthood, this naturally forces us to reinterrogate other factors at play, such as genetics. That these questions that Richard poses are not yet answered by the legal framework, I argue, stems from the fact that the law that exists was developed from principles that took biological human gestation in the person who intends to parent (with a uterus they were born with) as a given. The Human Fertilisation and Embryology Acts place assisted *conception* as the focus. Surrogacy was tacked on and not dealt with effectively when it became possible. The way surrogacy is treated in the law very much results from the fact that surrogacy is treated as unusual. Because of this focus on assisted conception in family formation, there has not been the same pressing need to engage with gestational elements that are considered material in attributing parenthood – working out how, why, and when they matter.

Many participants voiced strong opinions about the values underlining attribution of parenthood at present. For example, some participants, throughout their reflections, consistently criticized how biological links—both gestational and genetic—are culturally and legally emphasized in the legal framework for being cis-heteronormative.

Bobby: *“I don’t speak for all LGBTQ+ people – but…. I think the viewpoint that often is impressed on us is that it’s less than. It’s not real parentage or it’s not as meaningful or it’s not as legally or biologically as linked, and therefore it is not the gold star nuclear family rendition of creating a baby.”*

As Bobby explained, even where accommodations are made for same-sex female couples, for example in the HFE Act 2008, this is still treated as the ‘exception’ to the norm.

Participants reflected on the potential implications of a failure to address the bigger conceptual questions about attribution of parenthood in practical terms. There was some mention of birth certificates, and how appropriate labeling on them matters to parents (Auden, Bobby), but practical ramifications for parents were thought crucial. One participant explained the impact of affording parental responsibilities to individuals:

Bobby: *“just to pick a really common one like Vit*[amin] *K after the baby is born, are parents or the intended parents of that baby still going to be able to operate with autonomy over their baby? How is that going to be worded? Is that going to be dependent on biological links and, if so, then why?”*

What Bobby sets out here is a common problem in surrogacy because a surrogate’s consent to a parental order is not recognized as valid until 6 weeks after birth.^56^ The implication of Bobby’s comment raises the further interesting example of what the law would say about legal parents in a case where an individual seeks to reproduce using only donor material and gestation takes place, at least in part, outside of any body. This relates back to the fundamental question about what it is that matters for parenthood: intention, genetics, or gestation? Bobby’s point also really speaks to the fact that where the nature of gestation is shifted, there is some concern about the fact that the essentialism innate in the legal framework governing parenthood might result in a move toward essentializing genetic relationships (because there are no gestational ones). Another participant considered the reasons it is so important that there are clear rules about who the legal parent is and *when* they become a legal parent.

Ali: *“there’s a whole discussion around who’s responsible for what… It’s not just about medics deciding what you can and can’t do. It’s also you having a say on what can and cannot happen to, say, a fetus that is being gestated outside your body as well.”*

The concern here is a risk of excessive medical control over ex utero gestation^57^ and the wishes of putative parents being disregarded.

Relatedly to matters of legal parenthood, some participants reflected on questions about what information potential future children born following uterus transplantation or following (partial) gestation in an artificial placenta should be provided about *how* they were gestated (implying that gestation may increasingly be something that does not determine parenthood in the same way). While there has been long-standing debate about the importance (or not) of children being aware of their genetic origins,^58^ there has also been some recent interest in ‘the right to know one’s gestational origins’.^59^ So much so that the Law Commission and Scottish Law Commission, in their recent reform recommendations published in 2023, recommended the creation of a Surrogacy Register in which children born from Surrogacy could access information about the surrogacy arrangement surrounding their birth.^60^ There is already provision for children to have information about their genetic information when they are donor conceived,^61^ so this recommendation is really about recognizing the importance of information about gestational origins in the same way as also an important biological link. Because information about gestational origins has now been deemed important in the context of surrogacy by the Law Commissions, gestational origins where they relate to either a donated uterus or a machine (in the case of the artificial placenta, might be thought to be just as important to individuals.^62^ For some participants, knowledge of origins was seen as critical for children. In the first focus group, there was an interesting exchange in which participants elucidate the extent to which they see gestational ties as also about genetics:

Charlotte: *“Legal parenthood is obviously really important to me, obviously, but I think children having knowledge of all their genetics is really important to me. And that is, that belongs to that child and that child should have access to that information whether they are born through sperm donation, egg donation, surrogacy. They should have that information.”*

*Olivia: “And what about a [donated] uterus by the way-”*

*Charlotte: “Yes.”*

*Olivia: “That is not genetic.”*

*Alex: “It might have some genetic effects though. I think we don’t know how the epigenetic effects would play out.”*

These participants make the important observation that genetics and gestation are often discussed as if they are completely distinct, but they are not entirely.^63^ These additional and complex questions about gestational origins, what these are and how significant they are, should be encompassed within reflections about fundamental changes to the legal framework.

### III.B. Cis-Gendernormative and Heteronormative Assumptions in Parental Status

The second conceptual challenge to the legal framework of parenthood lies in addressing its gendered and heteronormative language and assumptions. Without doing so, some of the benefits of uterus transplantation and ectogestation that make it so appealing to marginalized groups, for example LGBTQ+ individuals, are limited.^64^

As Bobby put it, the law *“currently states whoever births the baby, regardless of genetic links, is the mother of the child. I wonder how that’s going to work…”* if/when more people who are assigned male at birth, or non-binary that were assigned female at birth, become able to give birth/gestate with forms of assisted gestation. They continue to ask *“*[a]*re we going to be afforded the same protections under the terminology of motherhood, because sometimes, it’s almost as if that terminology is being used as like a gold star… it feels almost as if that is worded to exclude folks who fall outside of the cis-heteronormative…”*. Auden added that this is already a live issue and that changing the gendered language around parenthood may be quite challenging because of how socially entrenched labels like ‘mother’ have become:

*“I know that* [gendered language is] *a massive issue at the moment in relation to appropriate naming of parents on birth certificates, for example, but I can also see lots of, sort of, pearl-clutching moral panic, I guess, about who is named what and how parental structures are defined in law…”.*

Auden’s observations reflect both the failings of the current legal framework and the contemporary political context in England and Wales. While the Gender Recognition Act 2004 represented some progress in enabling people to change their legal gender,^65^ there is a complete lack of recognition of nonbinary identities in the law.^66^ The Act is also explicit that changing one’s gender does not change one’s status as a mother or father of a child,^67^ which is a serious limitation for some individuals. The HFE Acts 1990 and 2008 similarly fail ‘to adequately accommodate trans individuals when ascribing parental status’.^68^ In 2020, *in R (on the application of McConnell),* the Court of Appeal affirmed that the person who gestates and births is the legal mother of a child irrespective of their gender identity. Consequently, a trans man was unable to be recognized as his child’s father causing considerable harm to both him and his child.^69^ The Court emphasizes the importance of certainty in the birth registration system as a reason for its decision, and the earlier high court decision had described the outcome as based on ‘common sense, common experience and the basic facts of life’.^70^

However, as Mahmoud and I have argued, this determination by the Court ‘obscures how “common-sense” assumptions are grounded in gendered, hetero-normative, and cis-normative stereotypes’.^71^ Brown describes how the decisions in both the High Court and Court of Appeal frame the matter as a technical and legal one, which enabled ‘the court to ignore some of the more conceptual questions and the issues of public policy that are undoubtedly raised by the underlying issue of the parental status of men who give birth’.^72^ Further, as Horn has observed, ‘the continued policing of the gender of gestational parenthood by social institutions not only causes tangible harm to individuals who are denied self-determination but also limits the possibilities for expanding or collapsing traditional ideas’ engrained within parenthood, pregnancy, and reproduction^73^—both socially and legally. Transphobic sentiment, particularly virulent in this jurisdiction, underlies the policing of gender and pregnancy.^74^ Consequently, trans individuals who wish to become parents have serious ‘difficulties in navigating [their] parenthood within a society in which they may face hostility towards their decision’.^75^ As Bobby explains:

[Re-evaluating the legal framework] *“could, as well, inadvertently, put trans folks again in the firing line, as we’re being debated as real human beings or not and whether we have the same rights or ability to raise and care for children as other people do. If you ever want to figure out what the public attitude would be, just go back 20 or 30 years and see what the public attitude was to same-sex families, accessing those same rights and protections. Because it’s just copy and paste, babe, it’s copy and paste…”.*

De-gendering the language around parenthood is necessary to help realize the promise of technologies like uterine transplants and artificial placentas. But how this is undertaken in a way that ensures that marginalized people are not targeted is, as Bobby noted, unfortunately, politically complex.

Tackling the two conceptual questions raised by assisted gestation—particularly the more novel practices, such as gestation within a person AMAB or even outside of the body, is no small feat. The exercise of overhauling the legal framework to be much clearer about what legal parenthood is, and should be, premised on will be a huge challenge. If a future legal framework is to be better attentive to lived experience,^76^ there must be consultation with potential service users of novel forms of assisted gestation:

Auden: *“…one of the spaces that I think would need a lot of careful work with people who are actually going to use the technologies, would be unpicking how some of the family law and parental assignment rights in, sort of, naming and things like that, is unpicked in a way that represents what feels appropriate to the people who are using the technology.”*

Hammond-Browning has suggested that ‘questions around legal parenthood must be addressed at a legislative level before [the advent of ectogestation] ... The continuing acceptance of the importance of legal parenthood requires forward thinking of the implications and ramifications’ of artificial placentas.^77^ She suggests a period of consultation and debate resulting in legislative reform, which is echoed by Adkins.^78^ This analysis echoes her call—but with two important caveats.

First, we need to think about whose voices are centered in consultation/debate about how to attribute parenthood. Specific reflections on public engagement are discussed in the final theme in this article. Here, I make the observation that more research is clearly needed to find out who the potential service-users of novel forms of assisted gestation are, and in what circumstances they may want to utilize these technologies. This is necessary in order to anticipate what some of the potential legal issues they may face are and whether the anticipated attribution of parenthood (based on the current legal framework) does not match their perception of parenthood when using novel forms of assisted gestation. The individuals who took part in these studies all have knowledge based on their own experiences and/or interactions with people who need assistance during reproduction (whether with conception, gestation, or birthing). However, this is not a substitute for qualitative research with people who identify themselves as potential service users of uterine transplantation or ectogestation.^79^ In this qualitative research care must be exercised to ensure that it is not only cis-heteronormative and other privileged experiences that are sought out.

Second, we need to ensure that reform to attribute legal parenthood is not confined to the question of a specific case (eg, gestational labor performed for by one person on behalf of another) but that novel technologies are used as a springboard for broader more fundamental reform to answer some of the fundamental questions: *why* does the law value gestational connections above all else in attributing parenthood? Is this the right approach? Richard stressed that it is important that this work interrogating the fundamentals of the legal framework is much broader than attempts made-to-date, which have resulted in only piecemeal reform that did not address the heart of the matter (for example, the introduction of parental orders in the HFE Act 2008).

Richard: *“I worry that it’s so big, what we need, that it won’t be done, and it will be done bitwise, and after the event. And there is so much that is quite fundamental, like what does ‘mother’ mean, what does ‘parenthood’ mean? De-gendering all of the language around it. And it seems a lot to ask, to hope that those things will be done pre-emptively. Or that they will just be a big fat no, because of the yuck factor and the tabloid factor, and it won’t happen at all.”*

There will inevitably be complicating political factors in the process of law reform. That said, as the reflections of participants illuminate, the project of re-evaluating the fundamentals of legal parenthood is necessary. Piecemeal reform in response to different technologies as and when they are developed has not, thus far, been a successful strategy. The role of speculation in regulation is addressed in the final theme of this paper, but here I note that, in clearly showing the issues with the current approach, and recognizing its failings to parents, those who help them reproduce, and children, participants’ observations have illuminated that we should consider broader reform that accounts for future responsibilities. This might involve fully interrogating the values (eg, gestational connection, genetic relatedness) by which we ascribe parenthood in detail, thinking about why we hold these as values, and imagining novel reproductive possibilities to understand the relative importance of these values.

## IV. RESISTING THE POLICING OF GESTATION

In both groups participants expressed concerns about legal rules creating hostile conditions for potential service-users of novel forms of assisted gestation and pregnant people more broadly. Concerns were expressed about the limited accessibility of novel forms of assisted gestation curtailing their capacity to provide reproductive support for marginalized groups. Equally, there was disquiet about coercive uses of the technology eg, pregnant people forced to use an artificial placenta rather than terminate a pregnancy. In combination, these concerns both speak to the policing of gestation—who is allowed to gestate, who is not, and in what conditions a person can cease gestating. These observations raise issues at the root of the law surrounding reproduction that need close attention.

### IV.A. Inaccessibility for Marginalized Groups

Concerns about assisted gestation being inaccessible to marginalized groups because of the operation of legal (and potentially intersecting extra-legal) barriers were raised in both groups.^80^ Participants did believe that the extra-legal barriers were the more material in terms of limiting access,^81^ however, they still identified a need for reform in the scheme surrounding assisted reproduction to ensure accessibility for marginalized groups. While no law dictates the exact circumstances in which people can access fertility treatment, nor is there any regulation stipulating who *cannot* access fertility services, the law still polices who can access assisted conception through the ‘welfare of the child’ clause.^82^ As Thomson explains, this clause ‘provides the clearest statement of appropriate recipient status’.^83^ The section stipulates that treatment services should not be provided to an individual unless ‘account has been taken of the welfare of any child who may be born as a result of the treatment (including the need of that child for supportive parenting)’, meaning that clinics must undertake a ‘welfare of the child’ investigation before they commence treatment.^84^ Uterus transplantation requires the use of IVF (since the transplant does not enable ‘natural’ conception),^85^ and so does complete ectogestation. In such cases, the clause would apply. Auden raised this concern:

*“[H]ow does privacy get written into how these, sort of, technologies are regulated or how the law works? I mean, I think maybe everybody else… would be more of an expert than I am in terms of how things, like the welfare of the child clause has been used historically through regulation, but, you know, as I say… my choice to conceive, gestate and give birth to my children was entirely private and nobody looked at whether I’d be any good at it, and so I think that question about how privacy is structured into it is, I think, a really important one…”.*

Auden’s observation illustrates that where assisted gestation necessarily involves assisted conception, this provision enables significant interrogation about people’s need for, or choice of, different approaches to reproduction. Questions might be asked about the reasons why people are (not) wanting to gestate as *part* of their assessment as a potential future parent. One of the key problems participants were concerned with about the operation of section 13(5) is the invasion of privacy and additional scrutiny that comes with being biologically and/or socially infertile. For many, the infertility journey is already fraught with uncertainty and the financial and social complexities of needing interventions to become a (biological) parent. They experience additional scrutiny because of the ‘explicit moral role that has been sanctioned for clinicians’ that enables them to gatekeep access to reproduction.^86^ That section 13(5) installs doctors as gatekeepers,^87^ and enables them to ask interrogating and personal questions about every aspect of lifestyle, is an invasion of privacy. Some commentators have raised their concerns about section 13(5) operating to discriminate against infertile people, since we do not and cannot impose the same kind of invasive assessment about whether people ought to reproduce on people who can reproduce without any technological assistance.^88^ Some participants also reflected on the way in which this provision seemingly entrenches inequalities of certain families:

Richard: *“[T]his phrase around ‘welfare of the child,’ which I obviously totally understand, I do also have a little bit of a difficulty with it because it is just another point of inequality. The amount of effort and attention and decision that non-cis-hetero people need to put in to becoming parents is tremendous and I think probably should be the thought that everyone should put into becoming parents, but welfare of the child is not asked in the same way, or prioritised in the same way, in cis-hetero parent pathways to parenthood. So, there’s an inherent prejudice, which maybe we’ll never overcome...”*

Here, Richard is highlighting that families outside of the cis-heteronormative are often subject to discrimination through additional interrogation. There is, thus, concern not just about ‘biologically’ infertile people being subject to more investigation but also ‘socially’ infertile people because of their sexuality. Bobby stressed that the interrogation of LGBTQ+ families undergoing fertility treatment is directly related to being made to feel that they do not fit the ‘gold star nuclear model’ of family building ‘*that obviously leads to people asking those questions, that they don’t cis-het folks that don’t require reproductive assistance of your right and your ability to parent. And all the background checks and stuff*...’.

There is academic concern about the use of section 13(5) to limit access to reproductive options, and the enabling of direct discrimination against diverse groups of people that could ensue. This academic commentary reflects many of the same concerns that participants highlighted. In the context of assisted conception, commentators have noted how this provision is conceptually incoherent^89^ and excludes marginalized people/groups.^90^ In work with Horn, I have highlighted that there are some interpretations of section 13(5) that could be operated to exclude of same-sex couples from using ectogestation.^91^ Elsewhere, Horn has noted that ‘traditional narratives of family and the gendered relations that structure those narratives are likely to inform who is allowed to use’ ectogestation.^92^ In the uterus transplantation context, O’Donovan has considered how section 13(5) might limit access for people seeking uterus transplantation.^93^ The observations of the participants in these focus groups – that raise section 13(5) as a potential tool of policing *who* can gestate or have access to technology that can gestate—reinforce the importance of the arguments made in the literature about the need to revisit and reform this provision. Participant observations reiterate that there is a need for greater interrogation about how choices about *gestation* could be evaluated.

### IV.B. Treatment of Pregnant People

Participants in both focus groups reflected on how options about gestation might impact on the choices available to pregnant people. Participants suggested that novel forms of assisted gestation might influence how pregnant people are treated and limit some of the existing options available to them: both in the matter of whether to gestate/terminate a pregnancy and in the regulation of their behavior during a pregnancy.

Abortion was not discussed in considerable detail in either focus group. However, it was raised in both as a healthcare resource that needs greater protection before advanced technologies assisting with gestation, particularly artificial placentas, are readily available. Auden mentioned that abortion must be considered because *‘there’s also then a question about how any law and regulation space* [introduced to address an issue arising with these technologies] *interacts with things like abortion law’*. It is interesting that abortion did not dominate discussion in either focus group (especially since academic debate about ectogestation has centered on abortion).^94^ This was likely in part because of the context of the conversation: ectogestation was on the table as on the spectrum of potential gestational reproductive options alongside surrogacy and uterus transplantation. The focus was thus on uses of these technologies as a reproductive choice for people who *want* to reproduce. It also likely speaks to the fact that many of the people in these focus groups did operate with the frame of reference as abortion as essential healthcare.

Alex and Faye did reflect on the potential of artificial placentas to threaten abortion access, because of the technology potentially enabling anti-abortion discourse (Faye) and perceptions about a shift in viability (Alex; Faye). This reflects the doctrinal and critical analysis in the literature. Several scholars have outlined that the construction of the Abortion Act 1967 means that abortion will remain accessible even if artificial placentas were to become widely available^95^—even if the medical perception of viability is changed as a consequence.^96^ However, there are, as participants highlighted, other threats: for example, legislative intervention to limit access following campaigns by anti-abortion groups, or an increase in conscientious objection.^97^ In a recent (but notably small) study, 41 per cent of Australian doctors indicated that an artificial placenta used at 22 weeks would change their opinions about abortion at this point in gestation.^98^ Commentators, however, have suggested that in England and Wales ‘an unregulated trend of doctors being increasingly uncomfortable with abortion as artificial placentas become more available’ is unlikely, because there are dedicated and established providers in this jurisdiction.^99^ That said, this last year (2022)—with the *Dobbs v. Jackson Women’s Health* decision in the US having ‘undone’ the recognition of a constitutional right to abortion^100^—has shown just how precarious abortion rights and access can be, even in high-income democracies.

Faye explained that abortion provision would feel more secure, in advance of this technology, if changes were made: *‘abortion needs to change, just decriminalise… the sort of idea that time limits… just scrap that’*. Decriminalization and the reform/abandonment of viability thresholds in abortion regulation are suggestions that have been made in the literature.^101^ While the focus should be on how novel forms of assisted gestation can help people who want to reproduce, there inevitably might be ethical issues arising in the context of all these technologies where some parties want to end a gestation and others do not. Decriminalization of abortion leaves more space for these matters to be considered sensitively and with any pregnant person centered in the decision-making, rather than the medical profession as the current legislative scheme demands.

In addition to the discussion about abortion access, there was also discussion about the impact on the freedom and agency of pregnant people. As Auden explained:

*[There is the] “important question about who has agency, who has responsibility, and what the law and the practical experience is around extracorporeal gestating outside the body. But I also think there’s a really interesting and tricky question about how that then impacts on people who are carrying a pregnancy and how choices are framed to them.”*

Faye raised similar concerns from a policy perspective:

*“I think we are seeing a big policy capture at the moment that is regulating behavior during gestation… now we are seeing more of, ‘well it matters what you do during pregnancy, it can impact on the fetus’. What you do during pregnancy is so important that we need to regulate it to such a high degree…* ”.

Both participants are explaining narratives of fetal risk in different ways. The second focus group explored how these narratives of risk, based on the pressures created by ‘good motherhood’ discourses, often *“are used to push and shape and frame and coerce – at different levels and in different ways –* [pregnant people’s] *decisions”* (Auden). In the first focus group, conversely, Faye reflected on how policies are informed by these narratives to coerce more directly in healthcare without any direct intervention from the law.^102^ Concerns have been raised in the ethics literature about the potential for extra uterum gestation to contribute to the ‘pathologisation of gestation,’ facilitating a culture in which gestation with more control is deemed preferable and bodies capable of pregnancy are seen as a threat to the fetus.^103^ While this was not raised by Faye or Auden as a direct legal challenge, there are ways in which there may be legal manifestations of the attempt to control gestation (and policy in healthcare often has the same coercive effect as law). There is a pattern within the case law of pregnant people being found incapacitated when they refuse surgical intervention in childbirth,^104^ and while the intervention necessary for extraction for ectogestation may be even more drastic than a cesarean section,^105^ the case law clearly evidences that the law has perpetuated the hyper-medicalization of pregnancy/birth.^106^ It is thus not implausible that, were partial ectogestation available, there could be a case in which it is claimed that a pregnant person lacks capacity, their pregnancy is potentially dangerous and the argument is made that it is in their best interest to have their fetus extracted for gestation *extra uterum*. The case law illustrates that the threshold for demonstrating incapacity is potentially treated as being lower in pregnant people.^107^ The fact that no judgment in cases where pregnant people deemed to lack capacity disagree with health professionals has ever gone against what health professionals have recommended (whether surgical intervention or medical management) as in a pregnant person’s best interest^108^ is cause for concern. As Auden suggested, we must think about how ectogestation is framed and presented. Presenting ectogestation as an ‘alternative’ to a complete pregnancy does have the potential to coerce pregnant people’s behavior.^109^ The framing of extracorporeal gestation in legal or policy terms will, undoubtedly, have a significant role in determining if/how this technology is a tool for coercion. Legal mechanisms that might facilitate coercive uses of ectogestation, even if they seem far-fetched, warrant further investigation. It seems naive to suggest it is possible to create legal framework for the regulation of technologies (that would inevitably have an impact on the way that pregnancy may be understood and treated) that is totally immune from the kinds of interpretation enabling coercive outcomes that contemporary legal frameworks are subjected to. Consequently, reflections about legal frameworks in the context of socially and medically reinforced narratives such as ‘good motherhood’^110^ that influence how the law is interpreted are important to consider. Many commentators have raised concerns about the ways in which novel reproductive technologies could come to place limits on reproductive freedom^111^ (with people compelled to continue pregnancies,^112^ or to limit who is considered an appropriate gestator,^113^ or to place limitations on how gestators’ behaviors and choices,^114^ or feeling like they have to make the choice to use technology in order to be treated equally in the workplace).^115^ These reflections are as much a matter of how any novel legal framework is interpreted by medical, social, and legal institutions, as much as how it is written.

## V. MAKING SPACE AND TIME FOR LAW-MAKING

There was consensus, as Yan explains, that.

“*the law is very, very outdated and not keeping up with the technology. This makes everyone confused. The clinic are confused, the public are confused, and the law makers themselves are confused.”*

Naturally, in reflecting on what legal reform might need to happen there was also discussion about *how* this might happen. The final theme constructed within the data relates to law reform and the evolution of the law as a process. Within this theme, there is reflection on the role of public education in the introduction of novel technologies and debate about the merits and limitations of proactive regulation of novel reproductive technologies.

Participants felt strongly that people working in the reproduction should be thinking about novel technologies/practices around the corner and on the horizon and sounding out problems and solutions.

Auden: *“[I]t strikes me that that is something that would need a lot of space and time for people who were going to use it to explore, rather than it trying to impose a regulatory or a legal structure that doesn’t match up to either the technological reality or the lived experience, and it being applied in this theoretical platform that is quite separate and not designed for this.”*

Auden continued to explain that sounding out potential problems by speculating was also important because the issues raised are not discrete: *“I have lots of questions about the interactions with other aspects of the reproductive space”*. This sense that academic speculative work is of value in considering future legal challenges, and that this is necessary to inform potential future regulatory solutions, is—unsurprisingly—a position shared by academics writing about novel forms of assisted gestation.^116^ Academic interrogation of future technologies is useful because it is not subject to the same limitations (often dictated by political concerns) about what is a priority, as is the case for other institutions engaged in the business of law reform eg, the civil service. There is, therefore, more time and space to consider future possibilities. Academic interrogation also makes space for a multitude of approaches and perspectives and for the collection of different forms of evidence.

Both focus groups reflected on the importance of public education about novel reproductive technologies as an essential element of thinking about future regulatory implications. As Hazel explained:

*“My view is that you always have to start talking about these things before they are a possibility to get people, you know, slightly warmed up to the idea and I don’t mean necessarily in favour but at least familiar with it so they are able to start discussing it. Because otherwise if everything is there all you know sort of wham bam then how on earth do they then try to think about in a way that isn’t a knee-jerk reaction…”.*

She elucidated that researchers and scientists find it difficult, and sometimes intimidating, to do public engagement work given the fear of *‘backlash’* resulting from misunderstandings about their intentions. Or, where there is public disagreement with the concept altogether. She continued:

*“I think that is the thing to understand, to be really clear about what the boundaries are and what the purpose is, why? I think that is always the thing, the public want to know, you know, in the story why people are doing this.”*

This is a particularly salient point in the contemporary context. In both focus groups there was disquiet about media narratives influencing public perception about reproductive technologies, who should use them, and in what circumstances. In the first focus group, participants noted that surrogacy had been consistently vilified by the media—except in those cases exemplified as exceptional and ‘moral’^117^—and this had infiltrated the public consciousness and thus perceptions about surrogacy. That several participants observed that this happens with surrogacy gives us a good indication of how uterus transplantation might be reported (and thus what the public might come to think of it):

Hannah: *“I can somehow see it* [uterus transplantation] *ending up being very villainised in the media until you have got sisters where that happens. And then all of a sudden it is a moral, deserving, altruistic thing that she has gone through all of this and all is hardy but she gave her sister her uterus. And then now it is all of a sudden okay… And it could just be because of the whole surrogacy thing where there is that case. Where it was, all of a sudden it is not Kim Cotton and, “Oh my God, baby selling,” it is a moral case of altruism between sisters and it is okay now because it is altruistic.”*

Indeed, the first case of uterus transplantation in England did involve sisters and this was something heavily emphasized in the reporting; this fact was frequently featured in the headline. The Guardian reported *‘Woman “over the moon” after sister donates womb in UK first’;*^118^ the British Broadcasting Service reported *‘Woman receives sister’s womb in first UK transplant*’;^119^ and the Daily Mail’s headline read ‘*First UK Womb transplant branded massive success as “selfless” mum, 40, donates uterus to childless younger sister…’.*^120^ The reporting was overwhelmingly positive and much of this is directly attributed to the sibling relationship between the donor and recipient (and also that the donor was already a mother herself). Hannah’s concerns about whether the reporting would be as positive if the facts were different are important given the varying motivations that people may have for wanting to donate.^121^ Public education could go some way to counteracting the potential sensationalist narratives that it is possible to imagine might be published about novel forms of assisted gestation. This is important because media ‘are key to the setting of agendas and focusing public interest on particular subjects [or aspects of them], which operates to limit the range of arguments and perspectives that inform public debate’ and perspectives.^122^ Fox, in her reflection on the changes made to the Human Fertilisation and Embryology Act in 2008, noted that what was, and was not, on the agenda for law reform was heavily influenced by the framing of the issues in the media, and media debates.^123^ The way that stories about novel forms of assisted gestation are told will be pivotal to the attitude toward social change, but also of law reform since public debate and perspectives can influence what reform is deemed politically palatable. Fox noted that we must learn from the story of reforming the HFE Act, which ‘suggests that in order to inject feminist values into the process of legislative reform, feminists need to become more media savvy’.^124^ Moreover, as some academics have posited that public consultation is a necessity in law reform projects surrounding novel reproductive technologies,^125^ education about the purpose of novel forms of assisted gestation and what they can (and cannot) do will be important to ensure that public opinion is not dictated by misinformation.^126^ While participants in this study did not make any observations for or against public consultation, the participants’ feelings about the media and about public education are central to the conversations about consultation. Public education and media reporting are critical aspects of the process of law reform even if there is no direct public consultation.

While participants agreed that public education/understanding mattered, there was far less consensus about the merits of proactive law-making/regulation of novel forms of assisted gestation. In the following exchange, two participants reflect on how failing to act proactively can result in piecemeal solutions to broader problems—namely, because issues are addressed through the judicial process or discussed in Parliament only *after* they have already become an issue for some individuals:

Hazel: *“It is difficult, isn’t it? I think the time it gets to court it is too late, you know, you are always after. You are nearly always after the fact, aren’t you? …*

Hannah: *“And about the CRISPR debates as well and like the moral issues that come out of like different types of editing with like germline editing obviously as seen as the “No, we are not going there,” until they do.”*

As Hazel and Hannah recognize, there has been only piecemeal reform surrounding reproductive technologies for a significant period (since the Human Fertilisation and Embryology Act 1990). As such, when a novel technology comes to fruition it is debated in isolation, and decision-making about what is to be done/what legal change is necessary only occurs when an immediate need arises. For this reason, scholars have argued for broader and more-encompassing reform, and reform that is speculative in the sense that it addresses novel possibilities that are not yet regularly used (uterus transplantation) or even yet possible at all (artificial placentas).^127^ While the exchange above does seem to show some sympathy toward proactive regulation, there was by no means consensus about this. Some participants were very much in favor of proactive regulation in theory, but were worried about the process:

Auden: *“I think we need an overarching framework of regulation… But how do you divorce the development of that from the – big P – Political situation, so that it is not used as a political football?”*

Other participants thought that law reform/proactive regulation might not lead to the best solutions, because regulating *before* the technology is in use does not allow time for the ‘issues’ in use to become apparent:

Richard: *“In terms of more invasive regulation, down at the level of operations if you like, I’m of two minds around that actually. I kind of think, in some ways, when something is new, you need to let it get in there and get messy for a little bit and then work out how best it should operate in some ways. And then in other ways, of course you need to be thinking about how do you protect the people involved.”*

The view on proactive regulation among both focus groups appeared distinctly less positive than it is in academic literature. However, the dominant position in the academic commentary and that of the participants in this study are more compatible than they initially seem if we look at the nuances. Richard, for example, was not specifically *anti-*proactive regulation but was expressing doubt that proactive regulation could be comprehensive until we have some idea what the use of these technologies looks like. He also raised the point that in letting things get ‘messy,’ there would need to be balancing in terms of ensuring that potentially vulnerable people involved are sufficiently protected in utilizing novel forms of assisted gestation. Thus, there might be some aspects of the technology that need to be legally addressed *before* novel forms of assisted gestation are used, but inevitably there would be other issues that cannot be anticipated or addressed before a period of use. Richard’s position is, therefore, that we need to make space for both proactive and reactive regulation. I think that most defenders of proactive regulation in the academic literature would not dispute this framing. No one is advocating for proactive regulation that is static and does not respond to what become the issues as technology is being used.

Both focus groups briefly considered the locus of reform or management of novel forms of assisted gestation in terms of the body responsible. As Yan explained:

*“I think one of the reasons that the law is very outdated is, I do not know, maybe there isn’t any specific body to look into this segment of the law. For example, if you’re talking about fertility and reproduction law, and there’s a group of you know taskforce called HFEA* [Human Fertilisation and Embryology Authority]*. They have regular meetings and talk about the law within reproduction, but there isn’t any other body that’s looking into surrogacy, uterine transplant, parenthood, and law.”*

This observation alludes to a potential need for a separate authority to the Human Fertilisation and Embryology Authority to be involved in the regulation of assisted gestation. This point would fit with the academic observations that, given the distinction in the ethico-legal issues that are raised by assisted *conception* and then assisted *gestation*,^128^ there may be some merit in regulating assisted gestation in a distinct (but complementary) regulatory framework.^129^ In the first focus group, participants had been reflecting on the complications of the role of the Human Fertilisation and Embryology Authority in the regulation of uterus transplantation and I then asked directly about whether the Human Fertilisation and Embryology Authority would want to be directly involved in necessary law reform for (and monitoring of) assisted gestation:

Facilitator: *“That is an interesting question though about the HFEA. Do we think they would want the power?”*

Olivia: *“No.”*

Alex: *“I think they are fed up at the moment.”*

Yan’s observation, combined with the group discussion about the Human Fertilisation and Embryology Authority not wanting additional spheres to regulate, adds to the case for a complementary, but distinct, framework and monitoring authority. As this is a novel idea, it merits further interrogation on the finer detail eg, how a legislative instrument could complement the existing law without making it harder for people needing assisted gestation to access it? That there are multiple readily applicable legal frameworks to novel forms of assisted gestation has been considered by commentators a cause for concern in the uterus transplantation context, for example, because the procedure would be regulated by the law surrounding human transplant and around assisted conception.^130^ As such, if a separate body for the regulation of assisted gestation was determined to be a good approach, exactly how this would intersect with the law surrounding assisted conception would need to be well thought through. It is for this reason that some other participants thought that the idea of a new framework at all was introducing too much complexity. As the reflections of participants highlight, this is an area that deserves more academic scrutiny in advance of the use of novel forms of assisted gestation.

## VI. CONCLUSION

These findings can inform current debates about law reform (currently focused on surrogacy), including the need to broaden the discussion to other forms of assisted gestation. As study participants described, there are synergies between surrogacy and other forms of assisted gestation. As Ali explained *“surrogacy today helps to build the case for it* [other forms of assisted gestation] *if you like, because we see what positive outcomes surrogacy delivers, and there’s a lot of parallels between what you’re talking about*.” While there is a growing body of legal literature examining novel forms of assisted gestation, this work tends to look at the technologies/practices in isolation, however, there is much to be learnt from considering the issues they raise collectively and using surrogacy as a case study. This is evident in how participants in this study used surrogacy as an example of potential failings of existing law and policy for uterus transplantation and artificial placentas.

This study uniquely considered the perspectives of reproductive rights advocates about novel forms of assisted gestation. These individuals have an important perspective from their work with service-users of reproductive technologies and in navigating the landscape of law reform in practice. This study, and its key themes, have evidenced the need for further consultation with affected parties and persons with unique knowledge about the challenges of assisted gestation in practice. The study adds important nuance to the literature, and it is encouraging that the analysis is orientated toward forward thinking about regulatory solutions to be informed by experience.

Significant reform of the law surrounding reproduction is necessary. The key themes in this paper related to areas of reform and how it happens. There is a need for a fundamental overhaul of the law surrounding parenthood to account for diversity in family formation and novel technologies and practices. We must also consider how existing regulatory and policy structures around assisted reproduction and pregnancy might impact potential service-users of assisted gestation, and to introduce reforms to improve access and protect individual agency. There is a clear role for speculation in these exercises, though exactly how proactive regulatory processes should be remains up for debate. In any event, this debate—grounded discussion about novel forms of assisted gestation—is urgent. Academic and policy reflections about law reform surrounding reproduction should be broadened, considering assisted gestation and the issues raised as a big picture, rather than only looking at individual issues, to avoid piecemeal commentary and reform.

## Footnotes

1 Mats Brännström and others, *Livebirth after uterus transplantation*, 385 Lancet 607 (2015).

2 These devices are sometimes referred to as ‘artificial wombs’—for why these technologies are more accurately termed ‘artificial placentas’ see Elselijn. Kingma and Suki Finn, *Neonatal Incubator or Artificial Womb? Distinguishing Ectogestation and Ectogenesis Using the Metaphysics of Pregnancy*, 34 Bioethics 354 (2020).

3 Seppe Segers and Elizabeth Chloe Romanis, *Ethical, Translational, and Legal Issues Surrounding the Novel Adoption of Ectogestative Technologies*, 15 Risk Manag. Healthc. Policy 2207, at 2208 (2022).

4 Partial ectogestation is the more imminent technological possibility: Elizabeth Chloe Romanis, *Partial ectogenesis: freedom, equality and political perspective*, 46 J. Med. Ethics 89–90 (2020); Elizabeth Chloe Romanis and Claire Horn, *Artificial Wombs and the Ectogenesis Conversation: A Misplaced Focus? Technology, Abortion, and Reproductive Freedom*, 13 Ijfab 174, at 177–179 (2020).

5 Virginia Braun and Victoria Clarke, *Using thematic analysis in psychology*, 3 Qual. Res. Psychol. 77 (2006); Virginia Braun and Victoria Clarke, *Reflecting on Reflexive Thematic Analysis*, 11 Qual Res Sport Exerc Health 589 (2019); Virginia Braun and Victoria Clarke, Thematic Analysis: A Practical Guide (2021).

6 Surrogacy might be better described as a process rather than a technology (since the gestational element is a ‘natural’ occurrence—it is the conception that is assisted by technology). I am thankful to Dr Nicola Williams and Dr Laura O’Donovan for raising this point in discussions.

7 Elizabeth Chloe Romanis, *Assisted Gestative Technologies*, 48 J. Med. Ethics 439, at 439 (2022).

8 Elliott Richards and others, *Uterus transplantation: state of the art in 2021*, 38 J. Assisted Reprod. Genet. 2251–2259 (2021).

9 Denise Mann, Good outcomes from first 5 years of uterus transplants, but concerns remain, https://medicalxpress.com/news/2022–07-good-outcomes-years-uterus-transplants.Html (accessed May 5, 2023).

10 Andrew Gregory, Woman ‘over the moon’ after sister donates womb in UK first, https://www.theguardian.com/lifeandstyle/2023/aug/22/first-womb-transplant-uk-hailed-massive-success (accessed August 29, 2023).

11 Emily Partridge and others, *An extra-uterine system to physiologically support the extreme premature lamb*, 8 Nature Communications 15,112 (2017); Harou Usuda and others, *Successful use of an artificial placenta to support extremely preterm ovine fetuses at the border of viability*, (2019) 221 Am. J. Obstet Gynecol 69.e1 (2019); Alex Charest-Pekeski and others, *Achieving sustained extrauterine life: Challenges of an artificial placenta in fetal pigs as a model of the preterm human fetus*, 9 Physiol Rep e14742 (2021).

12 For ethical analysis see: *Elizabeth Chloe Romanis, Artificial womb technology and the frontiers of human reproduction: conceptual differences and potential implications*, 44 J. Med Ethics 751 (2018).

13 Alex Charet-Pekeski and others, *Impact of the Addition of a Centrifugal Pump in a Preterm Miniature Pig Model of the Artificial Placenta*, 13 Front. Physiol. 925,772 (2022); See also *ibid.*

14 Emily Partridge and Alan Flake, *The Artificial Womb*, in Fetal Therapy: Scientific Basis and Critical Appraisal of Clinical Benefits (Mark Kilby and others eds., 2020).

15
*Supra* note 11.

16 Abby Larson and others, *The EXTrauterine Environment for Neonatal Development: Present and Future*, 25 Pediatr Dev Pathol 253 (2022).

17 John George, *Vitara Biomedical raises $25 M to advance its artificial womb technology for premature babies*, https://www.bizjournals.com/philadelphia/news/2022/08/09/vitara-biomedical-philadelphia-artificial-womb.html (accessed Aug 9, 2022).

18 Segers and Romanis, *supra* note 3, 2212–2213; Evie Kendal, *Form, Function, Perception, and Reception: Visual Bioethics and the Artificial Womb*, 95 Yale J Biol Med 371, at 376 (2022).

19 Elizabeth Chloe Romanis, *Artificial womb technology and clinical translation: Innovative treatment or medical research*? 34 Bioethics 392 (2020); M. Beatrijs van der Hout and others, *Interprofessional Consensus Regarding Design Requirements for Liquid-Based Perinatal Life Support (PLS) Technology*, (2022) Front Pediatr, http://dx.doi.org/10.3389/fped.2021.793531 (2022).

20 Romanis, *supra* note 7, 441.

21 There are some empirical studies in the literature about uterus transplantation that reports data from research with service users about their motivations, eg, Lisa Guntram, *May I have your Uterus*? *The contribution of considering complexities preceding live uterus transplantation*, 47 Med. Humanit. 425 (2021). There are also two empirical studies that consider how artificial placentas may impact on perceptions of abortion, one with the public and one with medical doctors: Leslie Cannold, *Women, Ectogenesis and Ethical Theory*, 12 J. Appl. Philo. 55 (1995); Lydia Di Stefano and others, *Ectogestation ethics: The implications of artificially extending gestation for viability, newborn resuscitation and abortion*, 4 Bioethics 371 (2020). However, these studies all have (appropriately) narrow focuses and do not consider broader questions about the regulation of assisted gestation.

22 Ethics approval was granted by Durham Law School Ethics Committee in April 2022. Written consent was obtained from all participants after they had received an information sheet about the study and had opportunities to ask questions. Participants were informed that they could withdraw consent for the data to be used up to one week after the focus group, and that they could end their participation in the focus group at any time.

23 Sue Wilkinson, *Focus group methodology: a review*, 1 Int. J. Soc. Res. Methodol. 181, at 186 (1998); Hannah Frith, *Focusing on Sex: Using Focus Groups in Sex Research*, 3 Sexualities 275, at 276 (2000).

24 Brett Smith, *Generalizability in qualitative research: misunderstandings, opportunities and recommendations for the sport and exercise sciences*, Qual Res Sport Exerc Health 137, at 141 (2018).

25 Romanis, *supra* note 7.

26 Braun and Clarke 2021, *supra* note 5.

27
*Id.*

28 Virginia Braun and Victoria Clarke, *Can I use TA? Should I use TA? Should I not use TA?*  *Comparing reflexive thematic analysis and other pattern-based qualitative analytic approaches*, 21 Couns Psychother Res 37, at 39 (2021).

29 Braun and Clarke 2019, *supra* note 5, 592.

30 Elizabeth Chloe Romanis, *Equality-enhancing potential of novel forms of assisted gestation: Perspectives of reproductive rights advocates*, 37 Bioethics 637 (2023).

31 Sections 36 and 42.

32
Ampthill Peerage Case [1977] AC 547, at 577.

33 Kirsty Horsey, *Legal Parenthood and Parental Responsibility*, in Family Law 304 (Ruth Lamont ed., 2022).

34 Section 33(1).

35
Banbury Peerage Case (1811) 1 Sim & St 153 H.

36
*Leeds Teaching Hospital NHS Trust v A* [2003] EWHC 259.

37 Human Fertilisation and Embryology Act 1990, section 28(6).

38 Section 37.

39 The agreed second female parenthood conditions are set out in Human Fertilisation and Embryology Act 2008, section 44.

40 Section 12.

41 Human Fertilisation and Embryology Act 2008, section 35.

42 Set out in the Human Fertilisation and Embryology Act 2008, sections 54 and 54A.

43 Kirsty Horsey and Sally Sheldon, *Still Hazy After All These Years*: *The Law Regulating Surrogacy*, 20 Med. L. Rev. 67 (2012); Rita D’Alton Harris, *Mater Semper Incertus Est: Who’s Your Mummy? * 22 Med. L. Rev. 357 (2014); Kirsty Horsey, *Fraying at the Edges: UK Surrogacy Law in 2015*, 24 Med. L. Rev. 608 (2016); Zaina Mahmoud and Elizabeth Chloe Romanis, *On Gestation and Motherhood*, 31 Med. L. Rev. 109 (2023).

44 For their reasons on the need for reform see: Law Commission and Scottish Law Commission, *Building families through surrogacy: a new law Volume I: Core Report,*  https://s3-eu-west-2.amazonaws.com/lawcom-prod-storage-11jsxou24uy7q/uploads/2023/03/1.-Surrogacy-core-report.pdf (accessed May 15, 2023), paras 1.10–1.14.

45
*Id, p*ara 2.11.

46 Law Commission, *Implementation of our reports,*  https://www.lawcom.gov.uk/our-work/implementation/ (accessed Jun 19, 2023).

47 I am grateful to Victoria Adkins for discussions on this point.

48 Emily Jackson, *Degendering Reproduction*, 15 Med. L. Rev. 346, at 347 (2008).

49 Elizabeth Chloe Romanis, *Regulating the ‘Brave New World:’ Ethico-Legal Implications of the Quest for Partial Ectogenesis*, (2020) (PhD Thesis, University of Manchester 2020), 231.

50
Amel Alghrani, Regulating Reproductive Technologies: New Horizons, 259 (2018).

51 I am grateful to an anonymous reviewer for encouraging me to verbalize this here. Some potential responses are explored and critiqued in my forthcoming monograph.

52 Horsey, *supra* note 33, 308.

53
*Id,* 325.

54 Benjamin Jones and others, (2019) *Uterine Transplantation in transgender women*, 126 Bjog 152 (2019); Alireza Hamidian Jahromi and others, *Uterine transplantation and donation in transgender individuals; proof of concept*. 22 Int J Transgend Health 349 (2021).

55 Here, Ali is talking about some of the unique benefits of uterus transplantation and ectogestation for individuals and groups. See Romanis, *supra* note 30.

56 Per the Human Fertilisation and Embryology Act 2008, section 54(7) and Adoption and Children Act 2002, section 52(3).

57 Victoria Adkins, *Impact of ectogenesis on the medicalisation of pregnancy and childbirth*, 47 J. Med. Ethics 239, at 241 (2021).

58 See debate about anonymous gamete donation: Lucy Frith, *Gamete donation and anonymity: The ethical and legal debate*, 16 Hum. Reprod. 818 (2001); and more recent discussion about mitochondrial donation: John Appleby, *Should Mitochondrial Donation Be Anonymous?* (2017) 43 J. Med. Philos. 261 (2017).

59 Andrea Mulligan, *Protecting Identity In Collaborative Assisted Reproduction: The Right To Know One’s Gestational Surrogate*, 34 Int. J. Law Policy Fam 20 (2020); Katherine Wade, *Reconceptualising the Interest in Knowing One’s Origins: A Case for Mandatory Disclosure*, 28 Med. L. Rev. 731 (2020).

60 In the Law Commission/Scottish Law Commission report they conclude ‘research also shows that the gestational period impacts development, and therefore, knowledge of one’s gestational origins could be important for the process of identity-formation,’: Law Commission and Scottish Law Commission, *supra* note 43, para 6.1. For a brief analysis see: Lottie Park-Morton, *Access to information: the Surrogacy Register and beyond,*  https://reformingsurrogacylaw.blog/2023/05/02/access-to-information-the-surrogacy-register-and-beyond/ (accessed May 15, 2023).

61 Human Fertilisation and Embryology Authority (Disclosure of Donor Information) Regulations 2004.

62 Romanis, *supra* note 7, 444.

63 Edith Heard and Robert Martienssen, *Transgenerational Epigenetic Inheritance: myths and mechanisms*, 157 Cell 95 (2014); Becky Mansfield, *Folded Futurity: Epigenetic Plasticity, Temporality, and New Thresholds of Fetal Life*, 26 Sci Cult (Lond) 355 (2017).

64 Romanis, *supra* note 30. See also Laura Kimberley and others, *Equitable access to ectogenesis for sexual and gender minorities*, 34 Bioethics 338 (2020).

65 There are serious criticisms of the Gender Recognition Act 2004 eg, how it perpetuates a mental illness model of transition: Andrew Sharpe, *A Critique of the Gender Recognition Act 2004*, 4 J. Bioeth Inq 33 (2007).

66
*R (on the application of Elan-Cane) (Appellant) v Secretary of State for the Home Department* [2021] UKSC 56. I am grateful to Dr Alan Brown for discussions about this.

67 Gender Recognition Act 2004, section 12.

68 Alghrani, *supra* note 50, 229.

69
*R (McConnell and YY) v Registrar General* [2020] EWCA Civ 559.

70
*R (on the application of TT) v The Registrar General for England and Wales* [2019] EWHC 2384 Fam, per McFarlane P at [133].

71 Mahmoud and Romanis, *supra* note 43, 121.

72 Alan Brown, *Trans Parenthood and the Meaning of ‘Mother’, ‘Father’ and ‘Parent’—R (McConnell and YY) v Registrar General for England and Wales [2020]* EWCA Civ 559, 29 Med. L. Rev. 157, at 169 (2021).

73 Claire Horn, *Artificial Wombs, Frozen Embryos, and Parenthood: Will Ectogenesis Redistribute Gendered Responsibility for Gestation?* 30 Fem. Leg. Stud. 51, at 68 (2022).

74
*Id;* See also Alice Margaria, *Trans Men Giving Birth and Reflections on Fatherhood: What to Expect?* 34 Int. J. Law, Policy Fam. 225 (2020).

75 Alghrani, *supra* note 50, 229.

76 Which is widely recognized as the major failing of the law surrounding surrogacy see Horsey, *supra* note 43.

77 Natasha Hammond-Browning, *A New Dawn: Ectogenesis, Future Children and Reproductive Choice*, 14 Contemporary Issues in Law 349, at 367 (2018).

78 Victoria Adkins, *The Warnock Report and Partial Ectogestation: Retracing the past to step into the future*, Med. L. Rev. doi.org/10.1093/medlaw/fwad008 (2023).

79 On the importance of empirical research see: Adkins, *id.*

80 Romanis, *supra* note 30.

81
*Id.*

82 Section 13(5) Human Fertilisation and Embryology Act 1990, as amended by Human Fertilisation and Embryology Act 2008, section 14(2).

83 Michael Thomson, *Legislating for the Monstrous: Access to Reproductive Services and the Monstrous Feminine*, 6 Soc. Leg. Stud. 401, at 410–411 (1997).

84 Human Fertilisation and Embryology Act 1990, section 13(5), as amended by Human Fertilisation and Embryology Act 2008, section 14(2); Horsey, supra note 33, 309.

85 Laura O’Donovan and others, *Ethical and policy issues raised by uterus transplants*, 131 Br Med Bull. 19 (2019); Laura O’Donovan, *Why uterine transplantation requires us to rethink the role of the pre-conception welfare principle*. J. Law Biosci. doi:10.1093/jlb/lsac028 (2022).

86 Thomson, *supra* note 83, 410.

87 Marie Fox, *The Human Fertilisation and Embryology Act 2008: Tinkering at the Margins*, 17 Fem Leg Stud 333, at 337 (2009); Rachel Fenton and others, *Finally fit for purpose? The Human Fertilization and Embryology Act 2008*, 3 J Soc Welf Fam Law 275, at 278 (2010); Ellie Lee and others, *Assessing child welfare under the Human Fertilisation and Embryology Act 2008: a case study in medicalisation*, 36 Sociol Health Illn 485, at 503 (2014).

88
John Harris, the Value of Life 150–155 (1985).

89 Emily Jackson, *Conception and the Irrelevance of the Welfare Principle*, 65 Mod. Law Rev. 176 (2002); Sacha Waxman, *Applying the preconception welfare principle and the harm threshold: Doing more harm than good?* 13 Med. Law. Int. 134 (2017); Sacha Waxman, *The Pre-Conception Welfare Principle: A Case Against Regulation*, (2018) (PhD Thesis, University of Manchester).

90 Sheelagh McGuiness and Amel Alghrani, *Gender and Parenthood: The Case for Realignment*, 16 Med. L. Rev. 261, at 268 (2008); Julie McCandless and Sally Sheldon, *‘No Father Required’? The Welfare Assessment in the Human Fertilisation and Embryology Act 2008*, 18 Fem Leg Stud 201 (2010).

91 Claire Horn and Elizabeth Chloe Romanis, *Establishing boundaries for speculation about artificial wombs, ectogenesis, gender, and the gestating body*, in A Jurisprudence of the Body 242 (Chris Dietz and others 2020).

92 Horn, *supra* note 73, 69.

93 O’Donovan, *supra* note 85.

94 Romanis and Horn, *supra* note 4.

95 Jackson, *supra* note 48, 362; Alghrani, *supra* note 50, 148; Elizabeth Chloe Romanis, *Challenging the ‘Born Alive’ Threshold: Fetal Surgery, Artificial Wombs, and the English Approach to Legal Personhood*, 28 Med. L. Rev. 93, at 116 (2020); Adkins, *supra* note 57, 241; Elizabeth Chloe Romanis, *Abortion & ‘artificial wombs’: would ‘artificial womb’ technology legally empower non-gestating genetic progenitors to participate in decisions about how to terminate pregnancy in England and Wales?* 8 J. Law Biosci  http://dx.doi.org/10.1093/jlb/lsab011. (2021).

96 Romanis 2020, *supra* note 95, 116; Romanis 2021, *supra* note 95.

97 Romanis 2020, *supra* note 95, 117.

98 Di Stefano, *supra* note 21, 377.

99 Romanis 2020, *supra* note 95, 116.

100
*Dobbs v. Jackson Women’s Health* 597 USC __ (2022).

101 Claire Horn, *Ectogenesis is for Feminists: Reclaiming Artificial Wombs from Antiabortion Discourse*, 6 Catalyst: Feminism, Theory, Technoscience 1 (2020); Elizabeth Chloe Romanis, *‘Is “viability” viable? Abortion, conceptual confusion and the law in England and Wales and the United States,’* (2020) 7 J. Law Biosci  http://dx.doi.org/10.1093/jlb/lsaa059; Claire Horn, *Abortion Rights after Artificial Wombs: Why Decriminalisation is Needed Ahead of Ectogenesis*, 29 Med. L. Rev. (2021).

102 In contemporary context, for example, there is considerable surveillance and monitoring of pregnant people to try and ensure that they behave in ways deemed acceptable during pregnancy. There has been a noticeable move in the National Health Service toward coercive policies intended to monitor and reduce alcohol consumption in pregnant people: Rachel Arkell and Ellie Lee, *Using meconium to establish prenatal alcohol exposure in the UK: ethical, legal and social considerations*, J. Med. Ethics. doi:10.1136/jme-2022-108,170 (2022).

103 Giulia Cavaliere, *‘Gestation, equality and freedom: ectogenesis as a political perspective*, 46 J. Med. Ethics 76 (2020); Elizabeth Chloe Romanis and others, *Reviewing the Womb*, 47 J. Med. Ethics 820 (2021).

104 Eg, *Re S (Adult: Refusal of Medical Treatment)* [1992] 3 WLR 8; Re MB [1997] 2 FLR 426 (CA); Re AA [2012] EWHC 4378 (COP); *GSTT, SLAM v R* [2020] EWCOP 4; NHS Trust v JP [2019] EWCOP 23. See: Sara Fovargue and Jose Miola, *Policing pregnancy: implications of the attorney-general’s reference (no. 3 of 1994)*, 6 Med. Law. Rev. 265 (1998); Sara Fovargue and Jose Miola, *Are we still ‘Policing Pregnancy?’* in Pioneering Healthcare Law: Essays in Honour of Margaret Brazier, (Catherine Stanton and others eds., 2015); Samantha Halliday, Autonomy and Pregnancy: A Comparative Analysis of Compelled Obstetric Intervention (2019a); Samantha Halliday, *Court-authorized obstetric intervention: Insight and capacity, a tale of loss*, in Childbirth, Vulnerability, and Law: Exploring Issues of Violence and Control, (Jonathan Herring and Camilla Pickles eds., 2019b).

105 Jackson, *supra* note 48, 363; Amel Alghrani, *Regulating the Reproductive Revolution: Ectogenesis—A Regulatory Minefield?* in Law and Bioethics 316–317 (Michael Freeman ed., 2008); Romanis 2021, *supra* note 92.

106 Elizabeth Chloe Romanis and Anna Nelson, *The Medicalisation of Childbirth and Access to homebirth in the UK: Covid-19 and Beyond*, 29 Med. L. Rev. 661, at 674 (2021).

107 Halliday 2019b, *supra* note 104, 179.

108 Romanis and Nelson, *supra* note 106, 676.

109 Romanis and others, *supra* note 103, 826.

110 Mahmoud and Romanis, *supra* note 43, 133.

111 Nina Roesner, *Beyond a Medicalized View of Reproduction: Recentering Pregnant People in the Ethics of Ectogenesis*, 23 Am J Bioeth 102, at 103 (2023).

112 Romanis and others *supra* note 103, 826.

113 Cavaliere, *supra* note 99; Lisa Campo-Engelstein, *Reproductive technologies are not the cure for social problems*, 46 J. Med Ethics 85, at 85 (2020); Roesner, *supra* note 111, 103.

114 Romanis and others *supra* note 103, 826.

115 Zeljka Buturovic, *Formula feeding can help illuminate long-term consequences of full ectogenesis*, 34 Bioethics 331, at 335 (2019); Victoria Hooton and Elizabeth Chloe Romanis, *Artificial womb technology, pregnancy, and EU employment rights*, J Law Biosci doi:10.1093/jlb/lsac009, at 8–10 (2022).

116 Scott Gelfand and John Shook, *Introduction*, in Ectogenesis: Artificial Womb Technology and the Future of Human Reproduction, 1 (Scott Gelfand and John Shook eds., 2006); Amel Alghrani, *The Legal and Ethical Ramifications of Ectogenesis*, 2 Asian J. WTO Int. Health Law and Policy 189 (2007); Amel Alghrani, *The Human Fertilisation and Embryology Act 2008: a missed opportunity?* 35 J. Med. Ethics 718 (2009); Alghrani, *supra* note 50; Romanis and Horn, *supra* note 4.

117 Participants noted that a sister being a surrogate for her infertile sister is often heralded as the ultimate ‘moral’ example of altruistic surrogacy in contemporary reporting.

118 Gregory, *supra* note 10.

119 Fergus Walsh, ‘Woman receives sister’s womb in first UK transplant,’ https://www.bbc.co.uk/news/health-66,514,270 (accessed August 29, 2023).

120 Lucy Elkins, First UK womb transplant branded massive success as ‘selfless’ mum, 40, donates uterus to childless younger sister in huge medical breakthrough after crucial 17-h operation—giving thousands of sterile British women hope, https://www.dailymail.co.uk/health/article-12,432,017/Britains-WOMB-transplant-Huge-medical-breakthrough-sees-mum-40-donate-uterus-childless-younger-sister-mammoth-17-hour-op-wants-babies-can.html (accessed August 29, 2023).

121 There are people who have indicated a willingness to become a living non-directed uterus donor for a variety of reasons eg, to help others or they feel they no longer need their uterus. See Ann-Moree Warren and others, *Live nondirected uterus donors: Psychological characteristics and motivation for donation*, 18 Am. J. Transplant. 1122 (2018).

122 Catherine Happer and Greg Philo, *The Role of Media in the Construction of Public Belief and Social Change*, 1 J. Soc. Political Psychol. 321, at 321 (2013).

123 Fox, *supra* note 87, 340.

124
*Id,* 341.

125 Hammond-Browning *supra* note 77; Alghrani, *supra* note 50, 46; Adkins, *supra* note 78.

126 Adkins, *supra* note 78.

127 Alghrani 2009, *supra* note 116; Alghrani, *supra* note 50; Romanis, *supra* note 7.

128 Romanis, *supra* note 7, 440.

129
*Id,* 445.

130 Alghrani, *supra* note 50, 189; Saaliha Vali and others, *Uterine transplantation: legal and regulatory implications in England* 129 Bjog 515, at 591 (2021).

